# High frequency of prospecting for informed dispersal and colonisation in a social species at large spatial scale

**DOI:** 10.1007/s00442-021-05040-4

**Published:** 2021-09-22

**Authors:** Daniel Oro, Juan Bécares, Frederic Bartumeus, José Manuel Arcos

**Affiliations:** 1 Centre d’Estudis Avançats de Blanes-CEAB (CSIC), Acces Cala Sant Francesc 14, 17300 Blanes, Spain; 2 SEO/BirdLife-Marine Programe, Delegació de Catalunya, 08026 Barcelona, Spain; 3 CORY’S-Investigación y Conservación de la Biodiversidad, 08016 Barcelona, Spain

**Keywords:** Prospecting, Dispersal, Colonization, Information, Range expansion

## Abstract

Animals explore and prospect space searching for resources and individuals may disperse, targeting suitable patches to increase fitness. Nevertheless, dispersal is costly because it implies leaving the patch where the individual has gathered information and reduced uncertainty. In social species, information gathered during the prospection process for deciding whether and where to disperse is not only personal but also public, i.e. conspecific density and breeding performance. In empty patches, public information is not available and dispersal for colonisation would be more challenging. Here we study the prospecting in a metapopulation of colonial Audouin’s gulls using PTT platform terminal transmitters tagging for up to 4 years and GPS tagging during the incubation period. A large percentage of birds (65%) prospected occupied patches; strikingly, 62% of prospectors also visited empty patches that were colonised in later years. Frequency and intensity of prospecting were higher for failed breeders, who dispersed more than successful breeders. Prospecting and dispersal also occurred mostly to neighbouring patches where population density was higher. GPSs revealed that many breeders (59%) prospected while actively incubating, which suggests that they gathered information before knowing the fate of their reproduction. Prospecting may be enhanced in species adapted to breed in ephemeral habitats, such as Audouin’s gulls. Interestingly, none of the tracked individuals colonised an empty patch despite having prospected over a period of up to three consecutive years. Lack of public information in empty patches may drive extended prospecting, long time delays in colonisation and non-linear transient phenomena in metapopulation dynamics and species range expansion.

**Supplementary Information:**

The online version contains supplementary material available at 10.1007/s00442-021-05040-4.

## Introduction

Animals explore space searching for resources such as food, shelter against predators, and mates. It is well-known that dispersal processes are common in animal populations and they have been selected to increase fitness by settling in the best patch for surviving and reproducing (Bullock et al. 2002; Clobert et al. 2012). Since patch suitability is variable in space and time, prospecting is a dynamic process that allows individuals to gather information on the environmental quality of a breeding patch and make informed decisions to either stay in their current breeding patch or disperse (Reed et al. 1999). This prospecting process is also crucial for determining a species’ distribution expansion range through colonisation processes and the dynamics of spatially extended metapopulations (Bowler and Benton 2005; Kokko and López-Sepulcre 2006; Clobert et al. 2009). Nevertheless, how the quality of information gathered for decision making at individual level affects metapopulation growth and dynamics remains little known (Lister 2014; Gil et al. 2018). Prospecting was first described for social birds at the scale of a single colony: individuals assessed the reproductive output of conspecifics as a clue to decide which patch of the colony offered the best chance for successful recruitment in later years (Cadiou et al. 1994; Danchin et al. 1998). Further observations were made in adult collared flycatchers shortly afterwards (Doligez et al. 1999), and a review highlighted that dispersal may be preceded by prospecting (i.e. the so-called informed dispersal). Some studies showed that prospecting does not always lead to dispersal (Ponchon et al. 2017) and that prospecting occurs also at larger spatial scales (Ponchon et al. 2013). Since prospecting was first noticed, there was a growing interest in assessing which were the ecological drivers influencing this behaviour. For instance, prospecting increases after perturbations (e.g. increase of predation, harsh weather) and deterioration of patch suitability (Ponchon et al. 2015a; Payo-Payo et al. 2017), but patch spatial configuration, life histories, sex, social status and social environment and its feedbacks have also been appealed (e.g. Martínez-Abraín et al. 2003; Dittmann et al. 2005; Mares et al. 2014; Hanski and Cambefort 2014; Oro 2020). Nevertheless, empirical data to assess the importance of each of the potential drivers at large spatial scales are still scarce (Votier et al. 2011; Péron and Grémillet 2013; Ponchon et al. 2015a; Campioni et al. 2017).

From an evolutionary perspective, philopatry is favoured over dispersal when environmental conditions are good and are temporally autocorrelated. Thus, information gathered in a patch is likely to be useful to reduce uncertainty and increase fitness prospects. Dispersal may be costly because it implies leaving the patch where the individual has gathered key information about the environment, such as location of both resources and threats (Bowler and Benton 2005; Bonte et al. 2012). However, the environment is not stable and variability in resource dynamics force organisms to reset decision-making about whether to stay or disperse. Theoretical models suggest that prospecting to inform dispersal decisions evolves when the costs of information gathering are low and when mortality is high during the dispersal process (Schjørring 2002; Bonte et al. 2012; Bocedi et al. 2012; Delgado et al. 2014). In social species, that information is not only personal (coming from the individual experience and performance) but can also be public at different patches. Even though the breeding performance of conspecifics has been considered the only source of public information to assess patch suitability (Danchin et al. 2004; Dall et al. 2005), several studies have found that the number of conspecifics (and heterospecifics sharing the same guild) can also be used as public information to decide where to settle (Oro and Ruxton 2001; Serrano and Tella 2003; Fernández-Chacón et al. 2013). This may be especially true at large spatial scales where individuals have a short lapse of time to assess patch quality using the breeding performance of conspecifics (Oro 2020). In summary, individuals of social species are likely to make dispersal decisions using private and public information (Boulinier et al. 1996; Pärt and Doligez 2003; Parejo et al. 2006; Kivelä et al. 2014). However, public information is not available in empty patches, dispersal cannot be informed and colonisation is riskier, although some benefits can be obtained compared to dispersal to an occupied patch (e.g. lack of density-dependence). Despite the importance of dispersal and its ecological and evolutionary consequences for metapopulation functioning, little is known about how often prospecting occurs, the relative frequencies of informed and non-informed dispersal. Little too is known about which drivers influence prospecting for deciding where and when to disperse including colonisation dynamics and range expansion at metapopulation scales (Delgado et al. 2014; Ponchon et al. 2015b).

Studying prospecting behaviour in nature is challenging especially for mobile species due to the large spatial scales needed for detailed individual monitoring over time. Some results have been obtained using marked individuals and monitoring their location at different breeding patches across time, but incomplete information on their reproductive status and performance, limits conclusions (e.g. Duerr et al. 2007; Henaux et al. 2007; Davis et al. 2017; Genovart et al. 2020). The most suitable method for those species is telemetry tracking to monitor individual movement and behaviour with accuracy (Votier et al. 2011; Ponchon et al. 2013, 2015a; Casazza et al. 2020). Here we study the prospecting and dispersal processes in social Audouin’s gulls using a long-term monitored spatially structured population at large scale and tracked breeding individuals. The metapopulation study has historically shown an uneven clumped distribution of breeding patches, with a single patch holding up to 73% of the total world population, before experiencing a collapse due to an environmental perturbation in the form of invasive predators (Oro 2020). As a consequence, the number of occupied patches dramatically increased from 5 to 24 over the years and this allowed us to explore the colonisation process, which is difficult to track in field studies (Payo-Payo et al. 2017). Despite the small sample size, we will assess the importance of private and public information for making the decision of either stay or leave (i.e. of being philopatric or disperser). We used population size as a proxy of the available public information at each patch of the metapopulation. Since patches with larger population sizes have historically shown higher mean breeding performance (Oro et al. 1996; Cam et al. 2004; Fernández-Chacón et al. 2013; Genovart et al. 2018; Oro 2020), we could not disentangle the influence of either the breeding success of conspecifics or their densities (as different sources of public information) on dispersal decision. Larger colonies are more protected against aerial predators and they likely have more information to share about resources. Thus, we cannot disentangle which type of public information (either breeding success or the number of conspecifics, or both) was used to make the decision about either stay or disperse. However, we expected that individuals breeding in larger colonies dispersed less than those breeding in colonies with small densities, and individuals dispersing did so to larger colonies, where public information was higher.

## Methods

### Study species and sites

Audouin’s gulls are long-lived colonial species (mean adult survival = 0.9), with a monogamous mating system and a bet-hedging life-history. Modal clutch size is three eggs, and adults incubate for ca. 28 days after the third egg is laid in late-April. Chicks are nidifugous and fledge after ca. 30 days of parental care and fledglings and adults have seasonal migration to distant wintering areas mostly in the western African coasts under the influence of the Canary current (Bécares et al. 2016).

**Fig. 1:**
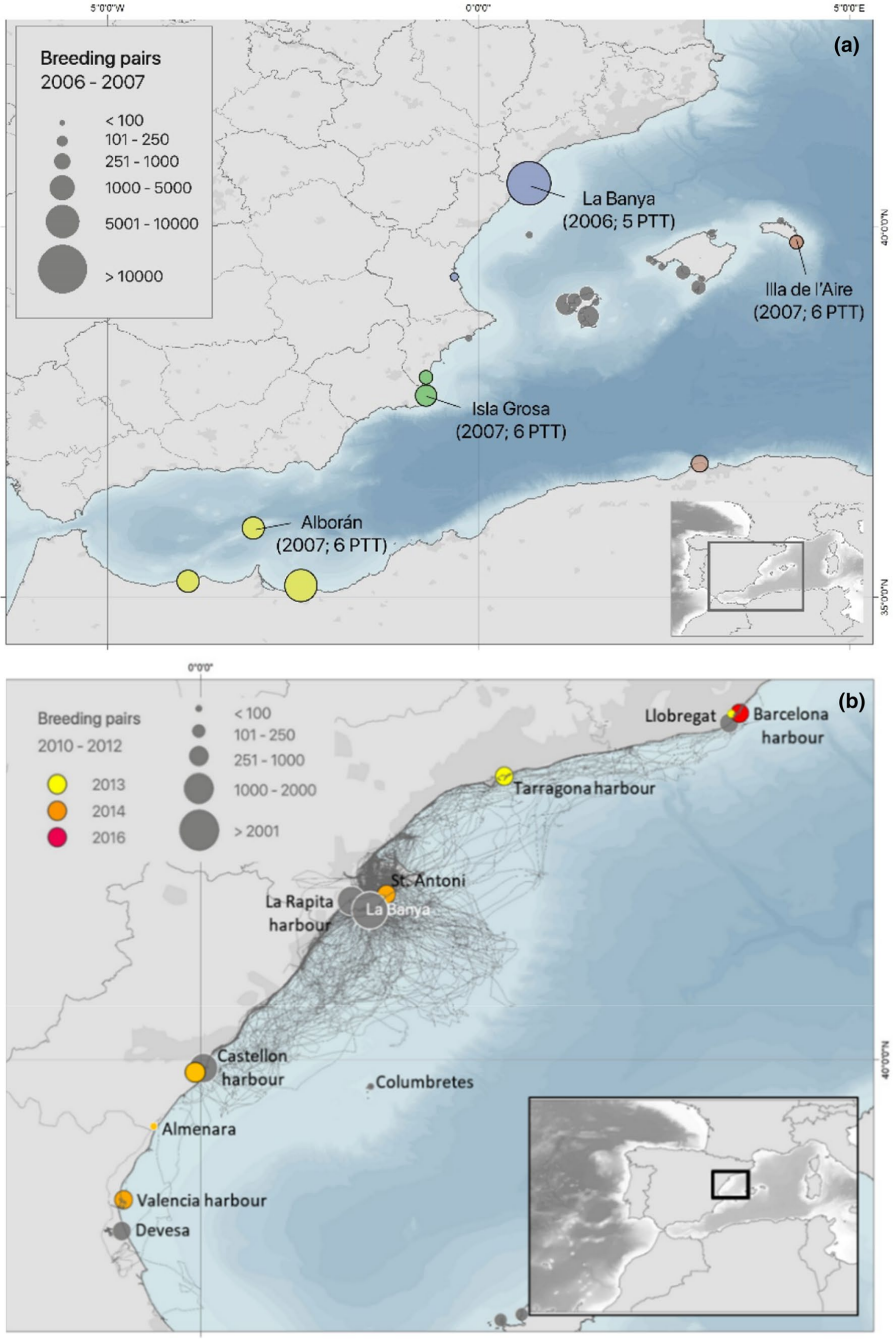
Map of the study area showing the patch spatial configuration including the study colonies where we tracked birds and the other patches (both OBP and FBP) at metapopulation scale. **a** Patches during PTT tracking (birds tagged in 2006–2007) showing the focal patches, the occupied patches (OBP, solid dots) and the empty patches (FBP, white dots) that were prospected during the breeding season; OBP that were not visited are shown as grey dots. Colours define sub-populations of focal patches with their prospected patches. **b** Patches during GPS tracking birds, all breeding at the La Banya (Ebro Delta) during 2010–2012, and trajectories for foraging and prospecting including OBP (in grey) and FBP (in different colours depending on the year of colonisation). None of the birds moved out of the area shown, during the breeding season. Size of dots for OBP show their population size during sampling

The western Mediterranean metapopulation holds 85% of the total world population. We have monitored population densities every year in all colonies of this metapopulation since the 80 s (Genovart et al. 2018) (mean number of occupied patches during 1981–2012 = 18; range of occupied patches: 7–26). Figure 1 shows the distribution of “occupied” breeding patches during the tracking [2006–2007 for platform terminal transmitters (PTT) and 2010–2012 for GPS tags] and “empty” patches that were colonised up to 5 years after tagging. Half of these colonisations occurred in harbours, a habitat never occupied by the species before. Breeding patch configuration changed over the years due to a perturbation occurring at the largest breeding patch at La Banya (Ebro Delta): in 1997, carnivores invaded and settled here. These carnivores (mainly foxes) have preyed on eggs, chicks and occasionally on adults, and they have also generated stress by their mere presence. It was not until several years later that many patches were colonised and La Banya experienced a rapid collapse to quasi-extinction (Payo-Payo et al. 2017; Genovart et al. 2018; Oro 2020). By colonized patches, we mean patches where gulls started to actively reproduce, building nests, mating, laying eggs and potentially raising chicks.

### Tracking birds

All birds were trapped at the nest during incubation and tagged with tracking devices (Table 1). In 2006–2007, 23 birds were captured at four different colonies (Ebro Delta, Illa de l’Aire, Isla Grosa and Alboran; Fig. 1) and tagged with 18 g solar PTTs, using a harness, which allowed for information for up to 4 years (Bécares et al. 2016). Later in 2010–2012 another 84 birds were captured at the Ebro Delta and tagged with 25 g GPS loggers (Table 1). Sex of birds was determined by body measurement (53% of males) but it was not included in the analysis due to the small sample size. For PTT tagging, selected data started at late incubation period of the first year (when trapped) until the device stopped emitting signals, for at least two consecutive breeding seasons. Fourteen PTT birds provided data for at least two of these seasons. Since GPS tags had to be recovered for data downloading, data corresponded only to the incubation period when birds can be trapped at the nest. PTT devices have poorer spatial resolution than GPS data and frequency was low (mean = 5 locations per day and individual during the breeding season from April to July, range: 1–19), but 14 birds yielded at least two consecutive years of data. These data allowed us to assess the role of prospecting for breeding dispersal. GPS data were retrieved for 46 individuals, which were incubating and were actively breeding until the tag was removed by trapping the birds again. The rest of GPS were not recovered due to our inability to recapture the individual, or because they provided no information due to technical problems with the device (i.e. sealing failure, download bugs). GPS data have much higher precision and frequency (every 5 min) than PTT, but tags were deployed for a maximum of 15 days.

**Table 1:** Remote monitoring data used in our study (year of monitoring in parenthesis) at each study colony (see Fig. 1)

Tracking device	Delta Ebro (15,329)	Aire Is (125)	Alborán Is (526)	Grosa Is (582)	Total
PTT	5 (2006)	6 (2007)	6 (2007)	6 (2007)	23
2-years of data	3	4	4	3	14
GPS	4 (2010) 36 (2011) 6 (2012)				46

Colony size (as number of breeding females) is shown in parenthesis for each breeding patch. For PTT, the number of individuals monitored over two consecutive seasons is also shown

Typically, trips performed by tracked birds during the breeding season corresponded to foraging bouts mostly at sea. For some but not all of these foraging trips, we observed gulls stopping at specific places along the coastline. In most cases, these stops occurred after a foraging bout on the way back to the colony. Out of the patches where gulls were breeding or colonised in later years, most habitats were not suitable for stopping due to intense coastal urbanisation. Two non-exclusive reasons may explain these stops out of the colony: resting and prospecting. Since tracked birds were actively breeding and constrained from returning to the colony regularly (where resting is a common behaviour), we assumed that these visits mostly corresponded to prospecting for information gathering. In other words, movements which did not occur at sea but along the coastline, most of the time after foraging on the way back to the colony were assigned as part of the prospection process, in which individuals can gather information for potential dispersal decisions. For PTT data, since devices yielded few points per day (maximum 15 points) and the spatial precision was lower (error encompassed several kilometres), locations within a radius of 7 km around the existing and future colonies were considered as prospecting visits. For GPS data, all stops taking longer than 10 min in a patch where gulls may potentially breed (other than the actual breeding patch) were considered to have been a visit for prospecting. We counted these stops as prospecting visits. In general, gulls stopped in only one patch other than their colony. For large breeding colonies, we delimitated the contours of different spatially discrete breeding patches, which were either occupied or not, in different years (Supplementary Figure S1) (Genovart et al. 2003).

### Analysis of PTT tags

The movements of each individual were analysed between April 15 and July 20 to determine whether it visited other patches and whether it failed reproduction, and how this affected whether and where it bred in the following season. Following field observations, we assumed that being absent from the colony for more than 4 days in a row away would indicate a reproductive failure (Oro 1998). We recorded the visits made by each individual to other breeding patches (Fig. 1a). Given the position error associated with the PTT system (Wilson et al. 2002), locations within a radius of 7 km around these colonies or future colonies (colonised up to 5 years after the tagging) were considered as visits. We assumed that a bird skipped breeding (i.e. took a sabbatical year) when it was not recorded in any colony (considering the PTT error) for more than 4 days in a row during the incubation and chick growth periods. From the 23 PTT tagged birds, 14 provided data over at least two consecutive breeding seasons (including the season when they were tagged) and allowed for the analysis of the role of breeding success and prospecting at year *t* on breeding dispersal at year *t* + 1. We considered it to be a breeding dispersal event when a gull was recorded settling in a patch during the breeding season other than the one used the previous season. We considered successful breeders (i.e. raising at least one chick) those who completed the breeding season visiting their focal breeding patch. We built GLM models to assess the factors affecting the occurrence of both dispersal and prospecting (as binomial response variables) and those affecting the intensity of prospecting measured as the number of visits to other patches (as Poisson response variable). For the occurrence of dispersal, we tested the explanatory variables of breeding failure and prospecting as categorical variables and the number of visits, and colony sizes (of both the patch of origin and the patch of destination) as continuous variable. We used the number of pairs counted at each patch as a proxy of the amount of public information available for gulls. For the occurrence of prospecting, we tested the explanatory variables of breeding failure and dispersal as categorical variables. For the intensity of prospecting, we tested the influence of breeding failure and the actual dispersal the year after on the number of visits per individual. Model selection was based on AICc values and two models with differences in AICc value < 2 were considered statistically equivalent (Burnham and Anderson 2002).

### Analysis of GPS tags

The movements of each individual were recorded with high spatio-temporal accuracy between May 13 and May 25 (corresponding to their incubation period) to determine whether the tagged individuals prospected other colonies or future breeding patches, which were mostly harbours. In total, gulls visited the following patches: Barcelona harbour, Llobregat, Tarragona harbour, La Rapita harbour, Castellon harbour and Devesa (Fig. 1b). Prospecting did not occur in colonies located within the maximum foraging range for an Audouin’s gull while breeding (180 km) (see also Arcos and Oro 1996): St. Antoni, Almenara, Columbretes Is. and Valencia harbour (Fig. 1b). For the analysis, we divided these patches into two types: (1) OBP, Occupied Breeding Patches (the exact location where there were active nests while tagged birds visited the patch); and (2) FBP, future breeding patches (where colonisation occurred up to 5 years after tagging). For each individual, we recorded the number of visits to each patch type and (3) time spent by individuals visiting each patch (1-h intervals). The difference in the frequency of prospecting animals visiting OBP and FBP was tested using the Fisher’s exact test. We performed an ANOVA analysis to test whether birds spent more time in OBP than in FBP. A likelihood ratio test was used to analyse whether birds visiting empty patches spent fewer nocturnal hours (when predation risk is higher) than birds visiting OBP. Finally, GLM models were built to assess the influence of distance to the Ebro Delta and population density at OBP on the occurrence of prospecting (as binomial response variable). This way, we tested the hypotheses that the occurrence of prospecting decreased with distance from the breeding patch and that the number of prospecting individuals increased with population density of the prospected patch, owing that larger colonies inform prospectors of more resources and for the study species, larger colonies have higher breeding success.

## Results

Considering all tagged birds, a large percentage (65%) prospected occupied patches, and 62% of these prospectors also visited empty patches that were colonised in later years.

### PTT tags

**Fig. 2:**
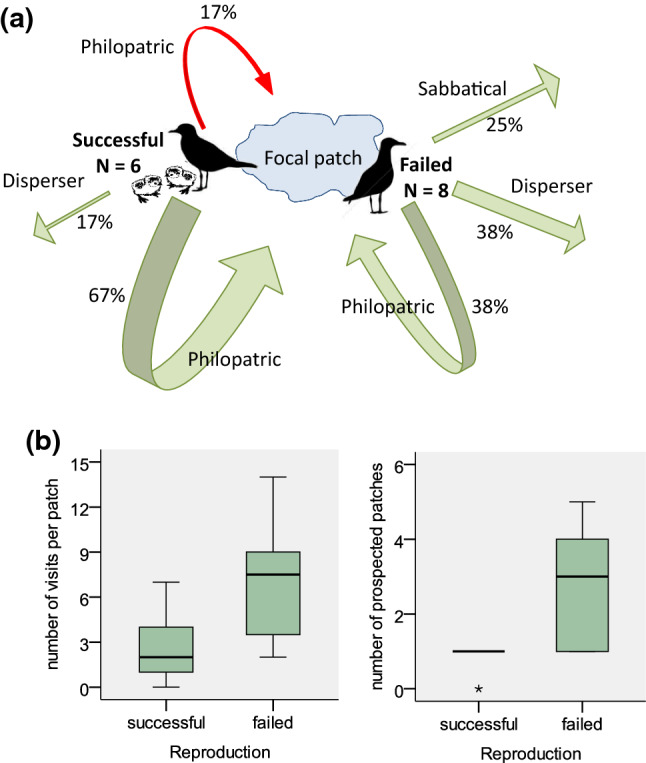
**a** Graphical representation of two consecutive years of prospecting and dispersal behaviour of 14 PTT-tracked gulls tagged in 2006–2007, depending on their breeding performance. The red arrow shows the only individual that did not prospect, whereas the rest of the arrows correspond to prospecting birds. The focal patch represents the breeding patch where the birds were marked (see Fig. 1a); **b** Box plots of the number of visits per patch and the number of prospected patches (considering both OBP and FBP) for successful and failed breeders (color figure online)

Figure 2 shows the dispersal decision made by the 14 individuals monitored over two consecutive breeding seasons depending on their breeding success and their decision either to stay (philopatric, 57% of the cases), to disperse (emigrant, 29% of the cases), or to skip breeding (sabbatical, 14% of the cases). Individuals tended to be philopatric after successful reproduction, whereas failed breeders made one of the three possible decisions: being philopatric, disperse or taking a sabbatical year. Most individuals prospected at least one of the monitored seasons (93% of total), and all failed breeders showed this behaviour. Interestingly, none of the four individuals breeding successfully and being philopatric the year after prospected other patches. The maximum distance recorded from a focal colony to an occupied breeding patch (OBP) of an active breeding individual was 164 km. However, one failed individual performed three trips from its breeding patch to a very distant patch 360 km away in northern Africa, where it dispersed the following season (Fig. 1a). The small sample size precluded most GLM models to be conclusive. Selected models for dispersal included all the factors tested except the size of the colony of origin and none of them was statistically significant (Table 2). For prospecting, the best model suggests that prospecting increased for failing breeders and with the size of the prospected colony but only breeding failure was statistically significant (GLM *z* value = 2.493, *P* = 0.013). On the contrary, the intensity of prospecting was significantly higher for failed breeders than for successful breeders (GLM *z* value = 2.886, *P* < 0.004) (Fig. 2b), whereas it was not statistically different between disperser and philopatric birds (GLM *z* value = 1.454, *P* = 0.146). The intensity of prospection increased with the population size of the prospected colony (GLM *z* value = 5.412, *P* < 0.001) (Table 2). The number of different patches prospected by the tracked gulls was also higher for failed than for successful breeders (*F*_1,13_ = 8.244, *P* = 0.014) (Fig. 2b). In four cases, individuals provided data for three to four consecutive breeding seasons (Table 3). Despite the very small sample size, results are consistent with what we observed for individuals tracked over two consecutive seasons. One of these cases corresponded to a gull that skipped breeding for two consecutive years before returning to its previous breeding patch.

**Table 2:** Models testing separately the factors affecting dispersal, prospecting and intensity of prospecting using data from PTT-tracked birds

	np	Dev	AICc	∆AICc	Wi
*For dispersal*					
Size_dest	2	15.203	19.203	0	0.20
Failing + Size_dest	3	13.871	19.871	0.668	0.14
Failing	2	15.992	19.992	0.789	0.13
Visits	2	16.427	20.427	1.224	0.11
Size_orig + Size_dest	3	15.206	21.206	2.003	0.07
Visits + Failing	3	15.211	21.211	2.008	0.07
Prospect	2	17.369	21.369	2.166	0.07
Failing + Size_orig	3	15.578	21.578	2.375	0.06
Failing + Prospect	3	15.589	21.589	2.386	0.06
Visits + Prospect	3	16.344	22.344	3.141	0.04
Size_orig	2	18.946	22.946	3.743	0.03
Failing + Prospect + Visits + Size_orig	5	15.068	25.068	5.865	0.01
Failing + Prospect + Visits + Size_orig + Size_dest	6	13.86	25.86	6.657	0.01
*For prospecting*					
Failing + Size_prosp	3	2.377	8.377	0.000	0.49
Failing	2	5.407	9.4067	1.030	0.29
Failing + Size_orig	3	5.986	11.986	3.609	0.08
Failing + Disperse	3	6.142	12.142	3.765	0.07
Failing + Disperse + Size_orig + Size_prosp	5	2.366	12.366	3.989	0.07
Size_prosp	2	15.924	19.924	11.547	0.00
Disperse	2	16.611	20.611	12.234	0.00
Size_prosp + Size_orig	3	15.099	21.099	12.722	0.00
*For the intensity of prospecting*					
Failing + Size_prosp	3	38.734	44.734	0.000	0.70
Failing + Disperse + Size_prosp	4	38.851	46.851	2.117	0.24
Size_prosp	2	45.740	49.74	5.006	0.06
Failing + Disperse	3	67.479	73.479	28.745	0.00
Failing	2	69.704	73.704	28.97	0.00
Disperse	2	77.825	81.825	37.091	0.00

*Visits* number of visits, *Failing* whether the individual failed reproduction, *Prospect* whether the individual prospected, *Size*_*origin* population size at the patch of origin, *Size*_*dest* population size at the patch of destination (for dispersal), *Size*_*prosp* population size at the patch of prospection (for prospecting), *np* number of estimable parameters, *Dev* relative deviance, *AICc* Akaike’s information criterion adjusted for small sample size (*c*), *ΔAICc* difference between current model and the model with the lowest AICc, *Wi* Akaike weight of model *i*

**Table 3:** Reproductive status of each of the four individuals (IDs) tracked with PTT tags for more than two consecutive breeding seasons depending on whether they disperse or stay as philopatric in their colony of origin (i.e. where they were tagged)

ID	Colony of origin	Year_*t*_	→ DA_*t*_	Year_*t* + 1_	→ DA_*t*+1_	Year_*t*+2_	DA_*t*+2_
33886	Aire Is	Failure (0)	Disperse (Devesa)	Sabbatical (55)	–	Sabbatical (31)	No more signals
59260	Grosa Is	Failure (21)	Disperse (Torrevieja)	Failure (1)	Philopatric	Success (3)	Philopatric
33888	Alborán Is	Success (1)	Philopatric	Failure (0)	Philopatric	Failure (4)	Philopatric
65796	Alborán Is	Success (0)	Philopatric	Success (5)	Philopatric	Failure (36)	No more signals

Numbers in parenthesis are the number of days prospecting other patches during the breeding season. *DA* decision after the breeding season in year *t*. Data for the two first years *t* and t + 1 are already included in Fig. 2 and Table 2. When an individual dispersed, the colony of destination is also shown in parenthesis

### GPS tags

**Fig. 3:**
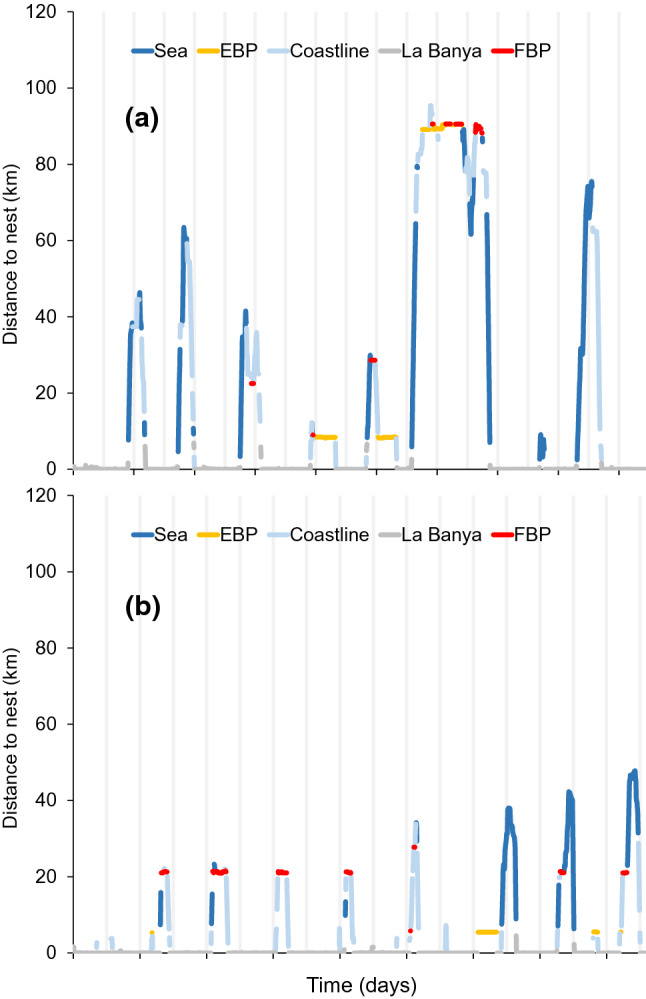
Two examples of GPS tracking data for individuals 5,107,916 and 5,107,927 (upper and lower panel **a** and **b** respectively) while breeding at La Banya. The graph shows the movements of these individuals over 9.5 consecutive days (vertical lines shows 12 h-intervals) corresponding to the late incubation period just before chick hatching. Consecutive points forming a horizontal line indicate no movement, which corresponded to stays either at the nest [for values of *Y* (distance to the nest) = 0], or prospecting (sometimes lasting up to 10 h) at other patches (OBP and FBP) (see Fig. 1b). Movements included several trips out of the colony, many of them to prospect

**Fig. 4:**
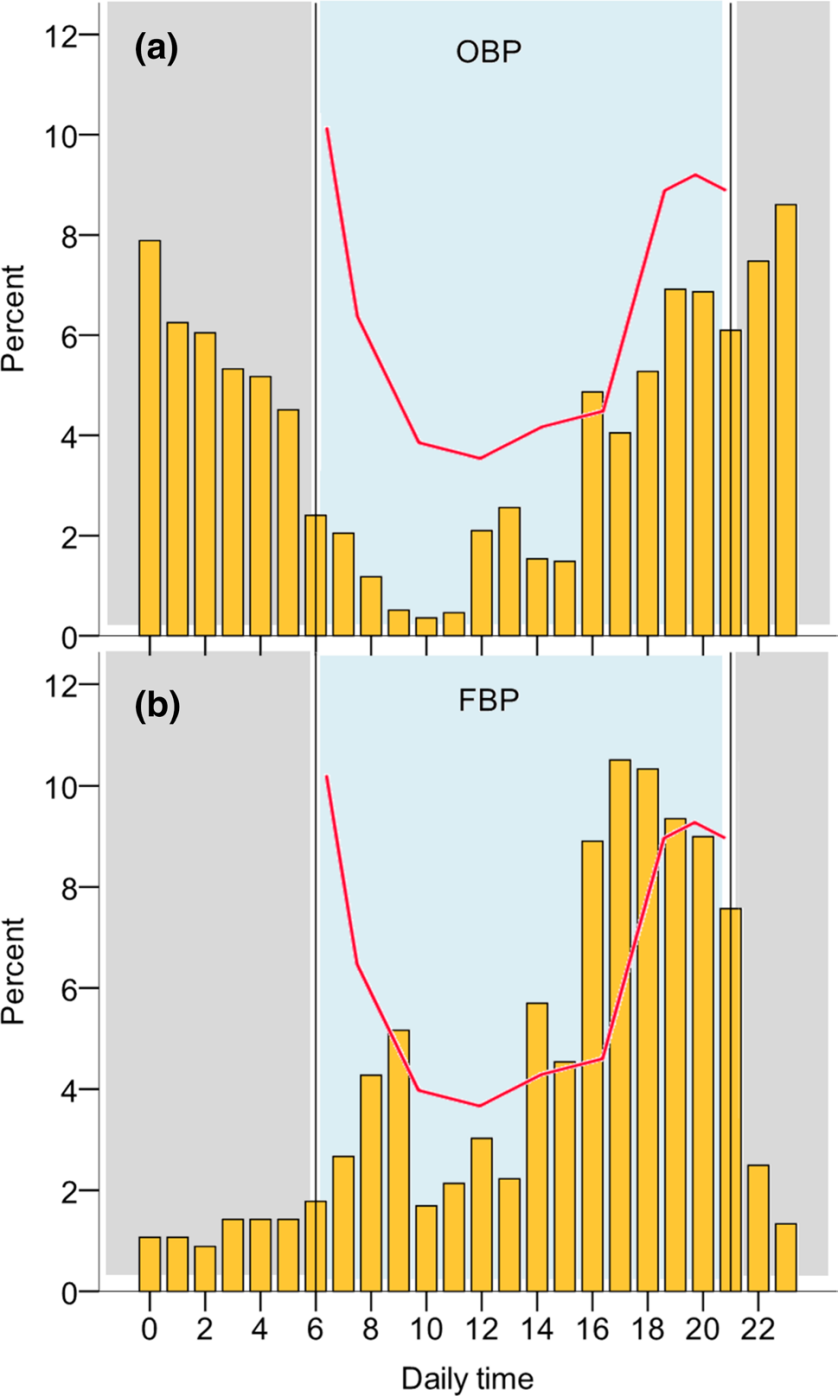
Percentage of GPS locations for birds breeding at La Banya (yellow bars) and prospecting established breeding patches (OBP, upper panel **a**) and patches that were colonised in later years (FBP, lower panel **b**) in the same colony (Castellon harbour, Fig. 1) depending on daily time. Red line shows the relative density of breeders at daylight time in an occupied colony ( adapted from Oro 1995) and is shown as a reference of the variation in density over that time compared to prospecting birds. The difference between patches was the presence of conspecifics in OBP (i.e. public information available). Data for the three sampled years are pooled

GPS tracking provided very accurate data on individuals prospecting the different patches at a large spatial scale during the breeding season (incubation period; Fig. 3). First, results show that 59% of birds prospected other patches before potential reproductive failure (i.e. while actively breeding): 50% prospected only OBP, 25% prospected only FBP (i.e. empty patches) that were colonised 1–5 years later, and 25% of birds prospected both occupied and empty patches (Table 4). The percentage of prospecting animals visiting OBP was higher than that visiting FBP, but the difference was not statistically significant (Fisher’s exact test, *P* = 0.290). The number of prospecting gulls per patch (including OBP and FBP) decreased with the distance to the focal colony (GLM *z* value = -2.444, *P* < 0.02) (Table 4). The number of prospecting gulls in OBP increased with population density at the patch (GLM *z* value = 3.401, *P* < 0.0001) (Table 4, Fig. 1b). Birds on average spent more time in OBP with breeding conspecifics than in FBP that were colonised in later years (*F*_1,68_ = 5.539, *P* = 0.021). Finally, birds prospected OBP and FBP at different times of the day: for OBP, the frequency of individuals prospecting at each daytime followed the same pattern of fluctuations in the number of birds present at colonies (Oro 1995, 1998), peaking at night, whereas birds visiting empty patches prospected at nocturnal hours significantly less (Likelihood Ratio test = 547.7, *df* = 1, *P* < 0.0001) (Fig. 4).

**Table 4:** Some descriptors of prospecting for the 46 GPS tagged Audouin’s gulls while breeding actively at the Ebro Delta

	Total	La Rapita	St. Antoni	Tarragona^a^	Columbretes^b^	Castellon^c^	Almenara	Llobregat^c^	Barcelona	Valencia	Devesa
Distance (km)		8	9	73	76	89	118	140	144	150	160
Population size		2155			68	973	25	355			671
Occupied breeding patches											
Prospecting gulls (and % from total)	20 (43)	12 (55)			0	6 (27)	0	3 (14)			1 (5)
Total number of visits (and mean value per gull)	41 (1.9)	26 (2.2)			0	12 (2.0)	0	3 (1.0)			1
Mean time spent per visit (hours)	4.5	2.9			0	8.5	0	2.7			0.2
Future breeding patches (year of colonisation)			2013	2013		2014			2016	2014	
Prospecting gulls (and % from total)	14 (30)		0	5 (31)		6 (38)^d^			5 (31)	0	
Total number of visits (and mean value per gull)	26 (1.8)		0	9 (2.3)		14 (2.3)			5 (1.2)	0	
Mean time spent per visit (hours)	3.3		0	3.5		2.6			4.7	0	

Patches within the maximum radius observed of gulls moving around the colony (180 km) are divided into occupied breeding patches (OBP) and future breeding patches (FBP), where colonisation occurred up to 5 years later (see Fig. 1b). The total number of prospecting gulls in OBP and FBP are single visits to different patches, and therefore, it is lower than the sum of this number at each patch (i.e. some gulls visited more than one patch). The distance of each patch from the Ebro Delta and their mean population size (as the number of nests), as well as year of colonisation for FBP, are also shown. Patches are ranked using the distance from the Ebro Delta colony. Data from the three sampled years are pooled

^a^Since this is a future breeding patch FBP, there is no value for population size

^b^This is the only island patch (not in the coastal mainland)

^c^This patch expanded its surface after the three years of sampling and contained both OBP and FBP

^d^These six individuals were the same as those that prospected OBP

## Discussion

Results from PTT-tracked Audouin’s gulls provide some insight on patterns on prospecting and breeding dispersal, although sample size was relatively small. First, breeding dispersal was much higher for failed breeders than for birds successfully breeding. Breeding failure is a very common driver for dispersal (Ronce 2007; Matthysen 2012), and personal information on individual own breeding performance is used to decide either to stay or disperse (Parejo et al. 2006). However, our results also suggest that this decision was fully made only after prospecting. All failed breeders prospected, and dispersal was more frequent for individuals prospecting more patches and performing more visits to these patches. This confirms the crucial role of prospecting to inform future dispersal events (Reed et al. 1999; Clobert et al. 2009). It is thus not surprising that prospecting is a widespread behaviour used by an individual to decide first if there is enough information to leave its patch and second, in which patch it would settle (Stamps 1988; Reed et al. 1999; Dall et al. 2005; Clobert et al. 2009; Schmidt et al. 2010; Morales et al. 2010). Prospecting would have been selected over evolutionary scales due to the potential benefits for fitness prospects, e.g. by lowering mortality (Kingma et al. 2016). These benefits result from gathering information (both personal and social) at alternative patches, which would reduce the potential risks of dispersal (Schmidt et al. 2010; Delgado et al. 2014; Ponchon et al. 2015b). Interestingly, we found that failed Audouin’s gulls gathering more information (i.e. prospecting several patches both occupied and empty) had more chances to disperse than those prospecting a single patch. At the scale of a single colony structured in spatially discrete sub-colonies, some previous studies on other social seabirds also show that individuals recruited at the most prospected sub-colony over the previous season (Dittmann et al. 2007). Further studies are needed to determine more precisely how the environment shapes prospecting in addition to all the other factors (e.g. occurrence of sociality, sex, personality, life-history strategies). Our results would apply for species occupying ephemeral habitats (e.g. coprophagous insects, species benefiting from fires and from unpredictable pulses of resources) and habitats with high spatio-temporal variability due to the stochasticity of physical drivers (e.g. marshlands, dunes, seasonal streams).

Even though we only analysed prospecting for breeding dispersal, previous studies on other species show that prospecting is also important for recruitment, i.e. the patch where individuals decide to incorporate to the breeding part of the population. Once again, recruiters may be philopatric (i.e. return to their natal colony to breed) or instead prospect other patches using public information (i.e. breeding success and the presence of both conspecifics and heterospecifics) to make natal dispersal decisions (Schjørring 2002; Ward 2005; Dittmann et al. 2007; Bosman et al. 2013; Casazza et al. 2020). For Audouin’s gulls, recruitment is mostly informed, meaning that it occurs more often when the individual has prospected the patch the year before (Genovart et al. 2020). Individuals recruiting without prospecting were all young first-time breeders affected by demographic carry-over effects, i.e. they were more reluctant to reproduce in subsequent seasons than individuals that recruited after prospecting. Prospecting in non-breeding birds has particular features compared to that performed by breeders. The former does not have energetic and spatial constraints imposed by reproduction, they have limited personal information (they have never bred before), and it seems that they mostly prospect late in the breeding season when public information on breeding success of conspecifics in different patches is available (Boulinier et al. 1996; Ward 2005; Greenville et al. 2016; Sherer 2019; Brandl et al. 2019; Tolvanen et al. 2020).

Our GPS results, using a larger sample size and higher spatial resolution, confirmed that prospecting in breeding Audouin’s gulls was a very common behaviour. Strikingly, this prospecting occurred while Audouin’s gulls were actively incubating, as it was found for Sandwich terns *Thalasseus sandvicensis* (Fijn et al. 2014). Contrarily, previous studies on social kittiwakes *Rissa tridactyla* found that prospecting among breeders only occurred after failing reproduction (Ponchon et al. 2015a, 2017). On the other hand, the occurrence of prospecting in Audouin’s gulls, as it was previously recorded for breeding dispersal, decreased with the distance from the focal patch (Oro and Pradel 1999; Fernández-Chacón et al. 2013; Bécares et al. 2016). At small distances, animals may revisit prospected patches to gather more information, to update information and to enhance informed dispersal, whereas distant patches require more sequential searches (Selonen and Hanski 2010). Furthermore, prospecting occurred more often in patches holding larger population densities, likely because they yielded higher public information and they indicate a large availability of resources (Cam et al. 2004; Fernández-Chacón et al. 2013; Payo-Payo et al. 2017; Genovart et al. 2020). Furthermore, results of Cam et al. (2004) on the study species did not support the hypotheses that dispersal was influenced by mean breeding success in the colony of origin (i.e. an indicator of habitat quality) or in the destination colony, or by the ratio of breeding success in these colonies (i.e. quality gradient). It seems that private information and colony size are the main clues used by Audouin’s gulls to make the decision about stay or leave the patch (Parejo et al. 2006; Fernández-Chacón et al. 2013). Studies dealing with other social birds also found that patches with higher population densities attract more individuals (Serrano and Tella 2003; Dittmann et al. 2005; Péron et al. 2010). Public information in the form of the number of conspecific at each patch is particularly conspicuous for social species since population density is a clue to readily assess patch suitability at large spatial scales (Nocera et al. 2006; Evans et al. 2016; Oro 2020). Some theoretical models highlight the role of density-dependence in breeding patch selection and of spatial heterogeneity in patch suitability (Nurmi et al. 2017).

### Drivers of prospecting

It was clear that Audouin’s gull changed their prospecting behaviour in empty patches compared to breeding patches where conspecifics were present. In occupied patches, prospecting visits overlapped with the numbers of breeders present, which suggest that prospectors look for higher density of conspecifics, either to increase information gathering or to increase protection against predation. On the contrary, prospecting birds avoided nocturnal time in empty patches, when the number of conspecifics to warn about the presence of predators is lower. Previous studies on the same species show that dispersal to occupied patches is performed mostly by younger breeders after a short, pulsed perturbation (Oro et al. 1999). Nevertheless, press perturbations (i.e. those that last several breeding seasons) may trigger colonizations of empty patches, and dispersal to these empty patches is performed by older, more experienced breeders (Payo-Payo et al. 2017, 2018). Despite our small sample size, our results also suggest that birds tend to disperse to patches where population density is larger, since these patches show higher breeding success. As more studies assess the occurrence of prospecting, it becomes clearer that it is a universal behaviour selected to reduce the costs of dispersal. Can our results on Audouin’s gulls be extrapolated to other species? Prospecting seems to be a *sine-qua-non* condition for dispersal, and drivers influencing the two processes would show a common pattern across taxa, but also specificities. First, the frequency of prospecting would decrease with patch stability (Schjørring 2002), since stability increases the temporal autocorrelation in patch quality (Danchin et al. 1998; Oro 2020). For instance, Procellariiformes are seabirds that breed in very stable habitats, and prospecting occurs mostly before recruitment, whereas once recruited, birds seldom prospect even after repeated breeding failures (Igual et al. 2007; Jenouvrier et al. 2008; Campioni et al. 2017). On the other extreme, species breeding in ephemeral habitats, such as Audouin’s gulls, terns and flamingos, would show the highest occurrence of prospecting (Erwin et al. 1998; Oro 2002; Oro et al. 2009; Acker et al. 2018; Francesiaz et al. 2020). Besides, the spatial configuration of patches, such as distance between patches and heterogeneity in quality, influences the occurrence of prospecting, which may be constrained by habitat loss and fragmentation (Schmidt 2017). The capacity of different species for prospecting at large spatial scales, especially in vagile species, may be higher than previously detected due to technological challenges and research biases (Cooper and Marra 2020). We found that one Audouin’s gull, after failing reproduction, was able to prospect at a very distant patch (360 km) where it dispersed the following season. We have mentioned that failing reproduction (or poor breeding performance) stimulates prospecting across taxa (Duerr et al. 2007; Ponchon et al. 2015a; Payo-Payo et al. 2017; Spendelow and Eichenwald 2018). Since breeding performance greatly depends on environmental stochastic conditions, harsher environment (e.g. habitat density-dependence, perturbations) would increase prospecting (Rémy et al. 2011; Payo-Payo et al. 2018; Sherer 2019). Nevertheless, prospecting may be increased in years of better environmental conditions, when physiological stress and costs of prospecting may be lower, as it has been recorded for social meerkats *Suricata suricatta* (Mares et al. 2014).

Besides environmental features, prospecting also varies within populations, depending on personalities (Schuett et al. 2012; Burkhalter et al. 2015), age, and sex (Doligez et al. 2004; Ward 2005; Wolfson et al. 2020), but data are still scarce to define a general pattern of their influence. Little is also known about the role of life histories, because even though most data come from long-lived species, short-lived species may also prospect (Pärt et al. 2011). Finally, social species have specific drivers of prospecting, such as social status and social environments (Mares et al. 2014; Kingma et al. 2016). In these species, recent studies show the importance of the social context of animals moving in groups and how decisions about where to go, to settle and to gather information would depend on what others decide (Mares et al. 2014; Strandburg-Peshkin et al. 2018; Oro 2020).

### Influence of prospecting on population and metapopulation dynamics

Since dispersal is mostly preceded by prospecting, prospecting may influence the local population and metapopulation dynamics (e.g. extinction-colonisation rates) (Delgado et al. 2011). Prospecting and informed dispersal also create asymmetric movements from degrading patches towards better patches, generating a heterogeneous distribution of individuals into the different patches (Ponchon et al. 2015b). In social species, many processes, such as information gathering and social copying, are density-dependent and generate feedbacks resulting in non-linear extinctions and population growth (Oro and Ruxton 2001; Gil et al. 2018). Individual decisions in social species partly depend on what others decide, and prospecting before leaving a patch and colonisation are processes that may generate non-linear transient phenomena in metapopulation dynamics (Hastings et al. 2018). In our study, none of the PTT-tracked individuals colonised a patch even though some gulls prospected empty patches for at least three consecutive years. Prospecting may last several years before deciding to settle after group dispersal (Morales et al. 2010; Munilla et al. 2016; Payo-Payo et al. 2017; Robinson et al. 2019; Oro 2020). Audouin’s gulls spent less time in empty breeding patches such as harbours, a novel habitat that was colonised in later years, and individuals gathered enough information for informed dispersal only after repeated visits over the years. Prospecting can be especially long when individuals explore novel environments where there is no public information about their suitability and this may lower colonisation rates that would ensure metapopulation performance and species range expansion (Schippers et al. 2009; Delgado et al. 2011; Schuett et al. 2012).

## Conclusions

It is assumed that animals move between patches to attain resources (e.g. food, shelter, and mates) for increasing fitness prospects. The other side of the coin is that stops between movement batches would correspond mostly to resting. Nevertheless, environmental stochasticity at spatio-temporal scales affects the amount of available resources and prospecting is a necessary process to gather and update information about the suitability of a patch. At ecological level, prospecting can also be useful when the environment is heterogeneous and is temporally auto-correlated (Danchin et al. 2004). Prospecting is a dynamic process requiring both movements for exploring space and stops for gathering information in a patch. Although prospecting at large scales has been explored for a limited range of species, it should increase for species evolving in very dynamic patches such as ephemeral habitats. Audouin’s gulls are social seabirds breeding in beaches and marshes that may change their patch availability and suitability from year to year. Our work shows that gulls prospect at large spatial scales while they are breeding, looking for suitable patches to disperse in case of conditions at the present patch would deteriorate. Although failing breeding promotes prospecting to increase fitness through dispersal, gulls having success also prospected, which confirms that gathering information is a key mechanism allowing organisms to make informed dispersal decisions (Clobert et al. 2009; Ponchon et al. 2015b). We show that stops at occupied patches were shorter than stops at empty patches, where public information is lack. In empty patches, information is gathered at a multi-annual scale and colonization of these patches would occur only after ensuring their suitability for breeding. Furthermore, an increase in prospecting may be a warning signal of deteriorating environmental conditions at the actual breeding patch. Since prospecting affects the ability to disperse by reducing the risks of leaving the actual patch, prospecting may affect metapopulation dynamics and the extinction-colonization turnover.

## Supplementary Information

Below is the link to the electronic supplementary material.

Supplementary file1 (DOCX 69 kb)

## Data Availability

The dataset supporting conclusion in this article is available at the Seabird Tracking Database of BirdLife International (http://www.seabirdtracking.org/).
