# Supplementary material for: High frequency of prospecting for informed dispersal and colonisation in a social species at large spatial scale
Source: Oecologia. 2021 Sep 22;197(2):395–409. doi: 10.1007/s00442-021-05040-4 (PMC8505276; doi:10.1007/s00442-021-05040-4)

Supplementary information

**Fig. S1**. Example of a site (a breakwater in the harbour of La Rapita) where gulls bred in spatially discrete patches (red surfaces) that were established in different years. Gulls may prospect established (i.e. with conspecifics) or future established patches (i.e. empty patches without conspecifics).


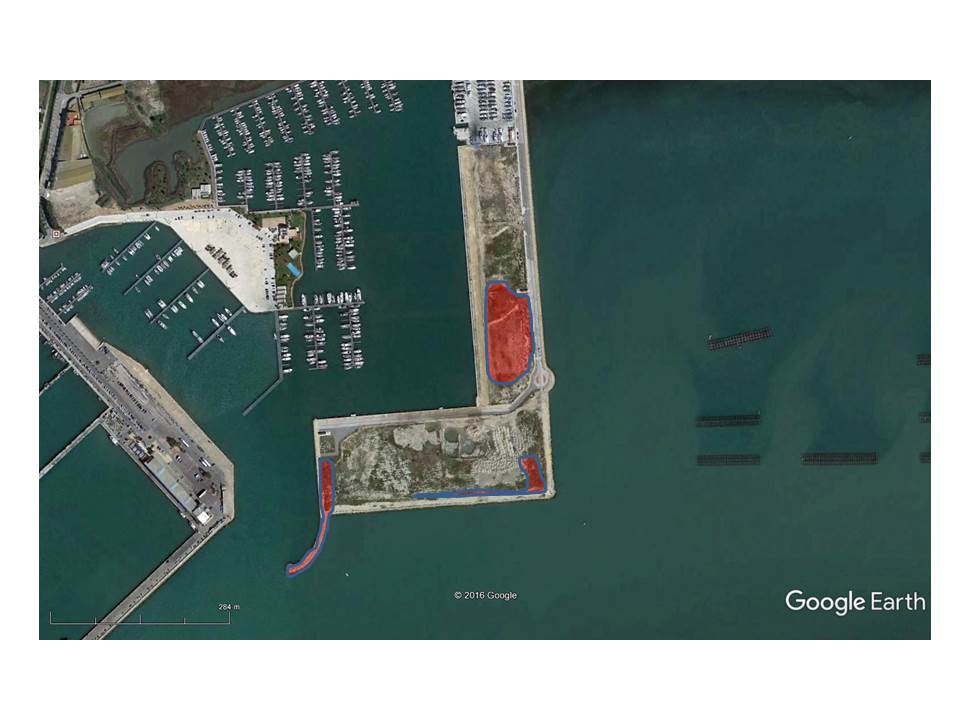

Supplement: Supplementary file 1 — Supplementary file1 (DOCX 69 kb) [file 442_2021_5040_MOESM1_ESM.docx]
